# Changes in retinal vessel oxygen saturation using automated retinal oximetry in patients with significant carotid stenosis

**DOI:** 10.3389/fneur.2025.1600749

**Published:** 2025-10-02

**Authors:** Petr Polidar, Barbora Pašková, Marta Karhanová, Martin Šín, Tomáš Mudroch, Tomáš Dorňák, Zuzana Schreiberová, Petra Divišová, Tomáš Veverka, David Franc, Petr Hluštík, Daniel Šanák, Tomáš Furst, Michal Král

**Affiliations:** ^1^Department of Neurology, University Hospital Olomouc, Olomouc, Czechia; ^2^Department of Neurology, Palacký University Olomouc, Olomouc, Czechia; ^3^Faculty of Medicine and Dentistry, Olomouc, Czechia; ^4^Department of Ophthalmology, Palacký University, Olomouc, Czechia; ^5^Department of Ophthalmology, University Hospital Olomouc, Olomouc, Czechia; ^6^Department of Ophthalmology, Military University Hospital Prague, Prague, Czechia; ^7^1st Faculty of Medicine, Charles University Prague, Prague, Czechia; ^8^Faculty of Science, Palacký University, Olomouc, Czechia

**Keywords:** ischaemic stroke, carotid ultrasound, retinal oximetry, arteriovenous difference, carotid stenosis

## Abstract

**Purpose:**

To evaluate the dynamics of changes in retinal oximetry parameters in patients with haemodynamically significant carotid stenosis.

**Methods:**

In this case-control study we examined 74 eye artery pairs in 37 patients with significant carotid stenosis over 50% using retinal oximetry. Fourteen eye artery pairs were excluded due to contraindications for oximetry or presenting carotid occlusion. From the remaining 60 eye artery pairs, 39 eye artery pairs formed the case group, remaining 21 pairs formed control group. Only 16 patients were eligible for bilateral testing to eliminate the possible influence of systemic comorbidities. Linear multiparametric model was used to predict the impact of the stenosis severity on retinal oximetry in context of systemic comorbidities.

**Results:**

Paired testing did not show any significant change in AV difference between stenotic and non-stenotic sides in 16 patients eligible for bilateral testing. The predictive model estimated weak correlation between increasing AV difference in increasing stenosis severity, with significant influence of atrial fibrillation and systemic atherosclerosis among noted comorbidities.

**Conclusion:**

The relationship between oxygen extraction in retina and increasing severity of the carotid stenosis is weak and modified by various individual factors, notably comorbidities.

## Introduction

Ischaemic strokes are the second most frequent cause of death and long-term invalidity, worldwide (1). Fifteen percentage of large vessel occlusions are caused by atherosclerotic stenosis of the internal carotid artery by over 50% (2).

While stenosis under 50% does not pose a significant risk of ischaemic stroke, in stenoses over 50% the significance increases. Asymptomatic hemodynamically significant stenosis over 50% has a 1-year relative risk of stroke of only 1.5 times higher compared to individuals with no stenosis and the risk does not differ between the stenosis severity of 50–69% and over 70% (3).

The ratio however greatly increases when presented with clinical symptoms. Symptomatic stenosis is defined as a stenosis with ischaemic event (amaurosis fugax, transient ischaemic attack—TIA—or minor stroke) up to 6 months prior to stenosis detection. Five-year risk of stroke recurrence in these symptomatic stenoses varies from 10% to more than 50% depending on other factors such as plaque characteristics, patient's age, type of symptoms and the time from the last symptomatic event (4).

### Treatment of carotid stenosis

Significant carotid stenosis can be treated via carotid endarterectomy. This one-time procedure can diminish the risk of ischaemic stroke to the level of a healthy person depending on concomitant risk factors. Compared to atrial fibrillation or small vessel cerebral disease which usually require life-long treatment with medication, this single-step procedure presents an unique opportunity to reduce one's risk of ischaemic stroke (5).

Despite the complex pathogenesis of atherosclerosis, only 2 criteria have proven to be clinically significant in making the decision whether to undergo surgical treatment. Those criteria are stenosis-severity and aforementioned prior clinical symptoms.

Clinical symptoms of the stenosis seem to be the key variable in predicting stroke recurrence and possibility of the devastating stroke event. However, classic symptoms—TIA, amaurosis fugax or minor stroke—are detectable in only 15% of cases (6). Eighty-five percentage of strokes diagnosed present as the first clinically apparent ischaemic event ever. So far, the only method capable of detecting clinically asymptomatic ischaemic strokes is MRI which is unsuitable as a screening method due to its prize, patient time burden and personnel and materiál requirements (7). Detecting the clinically asymptomatic yet subclinically active stenoses is now recognized to be crucial for stroke prevention.

### Automated retinal oximetry

Automated retinal oximetry allows for non-invasive evaluation of retinal blood vessel oxygen saturation and blood vessel diameter. These vessels are the terminal branches of the carotid basin. Retinal oximetry relies on the different absorption qualities of oxyhaemoglobin and deoxyhaemoglobin. While both oxyhaemoglobin and deoxyhaemoglobin absorb wavelengths of 570 nm, the absorbance of 600 nm wavelength is more prominent for deoxyhaemoglobin (8). The machine takes two monochromatic pictures of the retina. From the measured values of absorbance, it can mathematically extrapolate the arterial and venous blood oxygen saturation and arteriovenous saturation difference. Based on these parameters we are able to estimate retinal oxygen consumption. These parameters can provide information about the altered blood flow in the stenotic basin (9).

So far only one study has evaluated the changes in retinal oximetry in patients with significant carotid stenosis (10). This study included 16 patients with stenosis over 50%. Decreased retinal arterial diameter, increased arterial oxygen saturation and arteriovenous difference was observed in the stenotic group compared to both healthy controls and the contralateral nonstenotic side in the same patient using Wilcoxon paired testing. This testing compensated for interindividual variability of retinal oximetry results in patients with same severity stenosis due to systemic comorbidities as mentioned below.

The blood flow changes measured by ultrasonography are recognized as a golden standard method in evaluating stenosis severity. Ultrasonographic criteria for the estimation of the stenosis severity are described in Grant et al. (11) and rely mostly on evaluating the peak systolic velocity in the stenotic segment of the artery. Other parameters however such as plaque characteristics, residual lumen diameter, end-diastolic velocity or PSV-EDV ratio are used to help better evaluate the stenosis severity.

However, the estimation of the stenosis severity by carotid ultrasound is not simple. According to the continuity equation, the mass entering the stenotic segment must equal to the mass exiting the stenotic segment. This continuity is dependent on time factors, by which it differs from Hagen-Poisseule law commonly used to describe fluid dynamics. It states that in order for the flow volume to remain the same, reducing the vessel diameter results in increasing the flow velocity. Hagen-Poiseuille law usually describes laminar current of ideal fluid in rigid vessels which are not present in living human beings. Furthermore, in higher severity stenoses when peak systolic velocity cannot compensate for the stenosis, the blood volume in a certain time period is decreased. This actually results in reduced perfusion in higher severity stenoses in contrast to Hagen-Poisseule law. This also explains the discrepancy between the ultrasonographic assessment of blood flow velocities and the ischaemic potential of the stenosis. The accuracy of the stenosis severity prediction increases with its severity with the peak values between 80% and 95%. Due to the complex nature of the blood flow, lumen reduction under 40% might not present any haemodynamic changes (due to blood vessel pulsatility or systemic compensations). That is why sometimes there is a tendency to use the term “haemodynamically significant stenosis”—i.e., stenosis with the impact on the blood flow.

There are myriad other factors influencing the results of carotid ultrasound in one patient. One might be blood viscosity, other pulsatility and resistance of blood vessels especially considering the stenotic part caused by atherosclerotic plaque. These factors may differentiate individually in patients with the same comorbidity spectrum. Systemic factors, such as cardiac output or blood-flow demand might influence the ultrasonographic parameters as well. Individual variability of these factors might explain why in some patients PSV of 130 cm/s would represent 50% stenosis, while in another it might be normal non-stenotic blood flow velocity. Evaluation of carotid stenosis severity depends on assessing these factors individually and in spite of existence of radiological criteria might bear some clinical interindividual bias among physicians. Careful assessment of all aforementioned factors combined is necessary for optimal stenosis severity evaluation.

According to the results from Zhang et al. (10). The main underlying reason for the altered oximetry parameters in haemodynamically significant stenoses would be the result of the blood perfusion in the retina.

### Aims of this study

The aim of this study was to evaluate clinical relevance of altered parameters of retinal oxygen metabolism measured via automated retinal oximetry in patients with significant carotid stenosis. The relationship between the stenosis severity and increased oxygen extraction was already described in Zhang et al. (10) in a group of 16 patients. We aimed to analyze a larger number of patients, replicate the reliability of paired comparison between stenotic and non-stenotic sides in 1 patient.

Due to the limitations for the retinal oximetry evaluation we presumed bilateral testing would not be allways an option. In the second part of the study we strived to evaluate the relationship between increasing stenosis severity and retinal oximetry parameters in context of the systemic comorbidities that can modify the oximetry parameters. These comorbidities consist of diabetes mellitus, systemic atherosclerosis (coronary artery disease or peripheral vascular disease), chronic obstructive pulmonary disease, atrial fibrillation, congestive heart failure, smoking, alcoholism, hypertension, dyslipidemia and cognitive impairment (12–16).

There are several limitations of this study.

First, we noticed that only 16 patients (43.2%) were eligible for bilateral testing due to the contraindications for oximetry evaluation as mentioned below. For the patients ineligible for paired testing we strived to evaluate the relationship between oximetry parameters and stenosis severity in context of the presence of confounding comorbidities mentioned above. The impact of these comorbidities on retinal oximetry measurements is known, however its true extent in the general population or the effect of the combination of these comorbidities is not. Furthemore, the definitions of these comorbidities are potentially problematic especially in the systemic atherosclerosis diagnosis. While presence of the comorbidity is a weak parameter, study design not including biochemical analysis of the blood samples did not allow assessing the Framingham atherosclerotic risk score.

Second, there are certain conditions that exclude the possibility of oximetry evaluation as mentioned below in the methodology section.

Third–the low number of patients especially in higher severity stenoses groups. In higher severity stenoses the clinical manifestation of large ischaemic stroke is much more likely than in lower severity stenoses. These patients with significant neurological deficits often tend to be unable to undergo retinal oximetry testing. The real number of asymptomatic or mildly symptomatic patients with higher severity stenosis is very low.

The last limitation of the study is the absence of another method evaluating retinal blood flow, thus removing the possibility to compare retinal blood flow to retinal oxygen extraction. It is logical to presume that the change of retinal oximetry parameters would occur in the presence of altered blood flow. However, compensatory mechanisms underlying the stenosis formation consist of increasing the blood flow velocity in order for the blood volume to remain the same. Actually reduced blood flow can be observed in higher severity stenoses over 80% Therefore correlating the retinal oximetry parameters with stenosis severity itself might not be as optimal as correlating with actual blood volume reduction.

## Materials and methods

This case-control study was conducted between 2021 and 2023. Data from 37 patients with significant carotid stenosis over 50% were collected in the Neurology and Ophthalmology Departments of the hospital forming 74 eye artery pairs undergoing retinal oximetry evaluation. Mean age of the patients was 70.3 +/– 7.25 with female to male ratio 13:24. From his pool 4 patients presented bilateral stenosis forming the stenotic group of 41 eye artery pairs in the stenotic group and 33 eye artery pairs in non-stenotic group. Two eye artery pairs from the stenotic group and 12 eye artery pairs from the non-stenotic group were excluded from the study due to contraindications of retinal oximetry as listed below. The stenotic group then consisted of 39 eye artery pairs, 5 of them presented with retrograde ophthalmic blood flow. The 21 remaining non-stenotic eye artery pairs were used as a control group. In this way there were similar characteristics of the control group in terms of age, sex and cardiovascular comorbidity factors. Only 16 patients were eligible for bilateral testing comparing non-stenotic eye-artery to stenotic eye-artery as was done in Zhang et al. (10).

Sample characteristics are summarized in Table 1.

**Table 1:** Baseline sample characteristics, 39 artery-eye pairs in proband group, 21 artery-eye pairs in control group, 14 artery-eye pairs excluded due to contraindications for automated retinal oximetry.

**Number of patients**	**37**
Mean age +/- SD	70.3+/−7.25
Females (%)	13 (33.3%)
Case-control bilateral examination avaiilable	16 (43.2%)
Bilateral stenosis	4 (10.25%; 3 males)
Stenosis 50–69%	12 (30.1%)
Stenosis 70–79%	14 (35.9%)
Stenosis 80–89%	7 (17.9%)
Stenosis 90–99%	6 (15.4%)
Left sided stenosis	16 (41%)
Right sided stenosis	23 (59%)
Orthograde ophthalmic flow	34 (1 in group of 90% stenosis)
Retrograde ophthalmic flow	5 (1 in group of 80% stenosis, rest in 90% stenosis)
Diabetes mellitus type 2	17 (45.9%)
Chronic heart failure	13 (35.2%)
Systemic atherosclerosis (heart and periferal)	19 (51.4%)
Atrial fibrilation	10 (27.0%)
Hypertension	37 (100%)
Dyslipidaemia	37 (100%)
Cognitive impairment	2 (5.4%)
Chronic obstructive pulmonary disease	4 (10.8%)
Smoking	8 (21.6%)
Alcoholism	4 (10.8%)

Two in stenotic group, 12 in non-stenotic group. Graded age, sex, major comorbidities, % stenosis and collateral circlation.

The patients selected were both inpatient and outpatient. Both groups consisted of patients with either known stenosis of certain severity, or newly discovered stenosis. Ultrasonographic investigation took place prior to the retinal oximetry examination in the Neurology Department Neurosonology Laboratory.

An extended ultrasonographic protocol was used to determine the severity of the stenosis according to the diagnostic criteria given in Grants Radiology Guidelines (16). Multiple factors were used to assess the severity of the stenosis depending on patient anatomical differences, with the in-stenosis PSV and in-stenosis/post-stenosis PSV ratio as the key factor. Examination was carried out on the machine GE-Logiq-S8, linear probe 9L-D frequency 2–8 MHz, sector probe M5S-D, frequency 1.8–4.4 MHz.

The patients were referred to the Ophthalmology Department, where they underwent detailed ophthalmological examination including history of ophthalmologic diseases, physical examination, assessment of visual acuity, front segment on the slit lamp, optic coherent tomography with retinal nerve fiber layer measurement and automated retinal oximetry.

Examinations preceding the retinal oximetry were used to exclude patients with diseases ineligible for oximetry. These included high-degree refractive error +/- 6 dioptria, advanced cataract or glaucoma due to the inability to obtain pictures of adequate quality rate with cut-off value over 6/10. Furthermore, patients with diabetic retinal changes like proliferative angiopathy and the exudative form of macular degeneration or treated with anti-vascular endothelial growth factor drugs undergoing laser photocoagulation or pars plana vitrectomy had to be excluded as well (17–21).

The retinal oximetry examination took place in standard conditions in a dark room, eliminating visible light spectrum bias. A fundus photograph was taken at 50° width, centered on the temporal edge of the optic nerve. Flash intensity 50 Ws was used. At least 2 photographs of the same eye were taken. The higher quality photograph was later used for analysis.

The quality of the fundus photographs was evaluated automatically by software and only images with values exceeding 5 out of 10 were considered eligible. The quality ratio for the control group reached 7.938 +/– 0.549, for the stenosis group 8.0128 +/– 0.5884.

**Figure 1:**
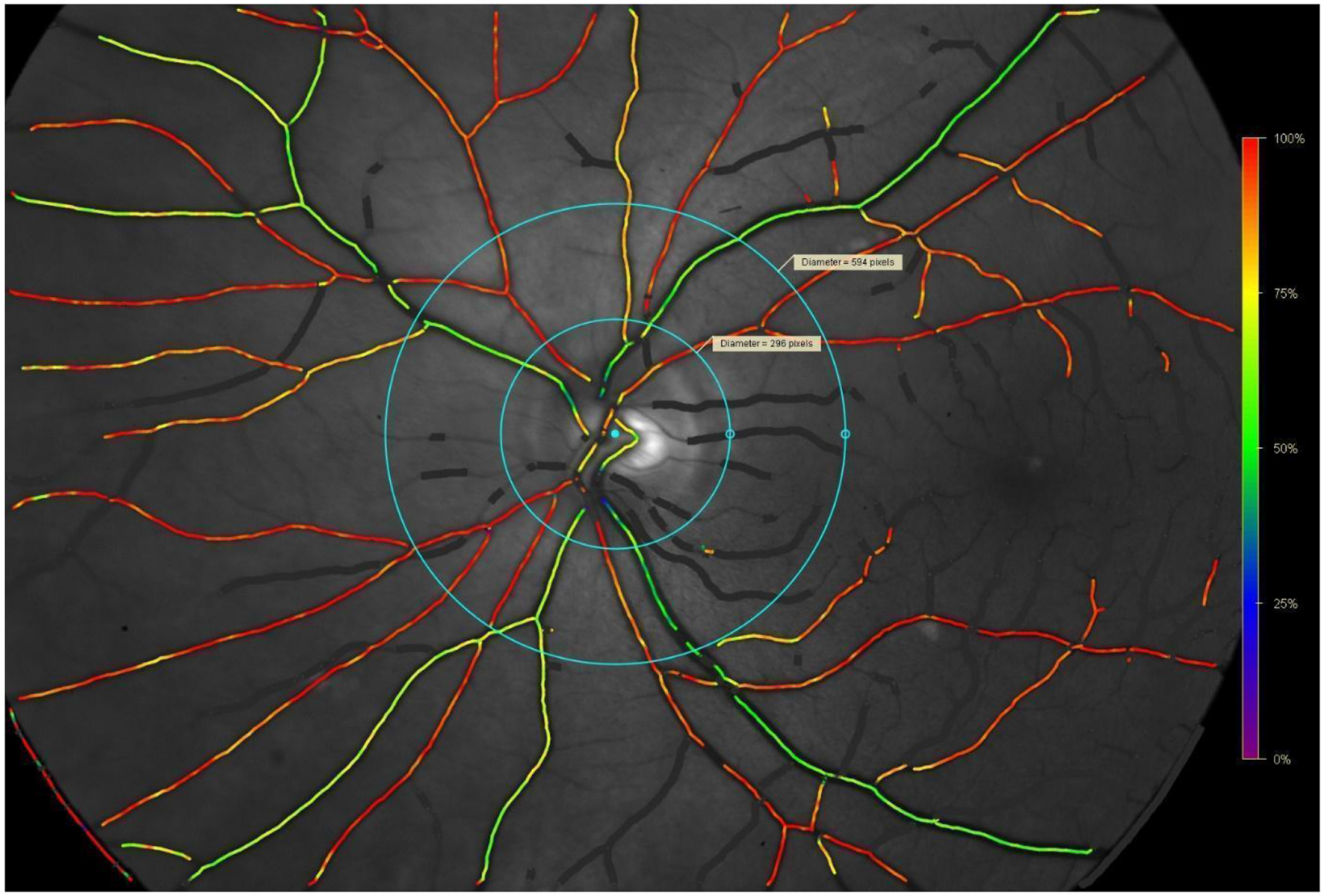
Example of retinal oximetrry photograph analysis. Dimensions: 100 mm (W) × 225 mm (D) × 350 mm (H). Angle coverage 50°. Measured wavelengths 5,700 nm (isobestic) and 600 nm (oxygen sensitive). Image dimensions 1,200 × 1,600 pixels. Standard deviation in 2 measurements of 1 blood vessel. Arteioles–SpO_2_ 0.8%, diameter 0.43–0.65 pixel. Venules-SpO 21.3%, diameter 0.46–0.60 pixel. Standard deviations describet at Palsson et al., IOVS 2012. Blondal R. et al., Graefes Arch Clin Exp Ophtalmol 2011.

The measuring area was marked by 2 concentric circles in 1.5 papillar diameter (PD) and 3PD, focussed on the center of the optic nerve papilla. The minimal resolution threshold for distinguishing the blood vessel was set to 6 pixels of vessel thickness, in our study sample minimal vessel thickness observed was 8 pixels−1 pixel corresponding to 89 micrometers approximately. Minimal length of a vessel segment was set to be 50 pixels, if the branching was present, main stem was excluded and summarized total length of all branches necessary for the assessment was 50 pixels. Four segments of retina in the region of interest were analyzed-superotemporal, superonasal, inferonasal and inferotemporal, with each segment containing at least 1 blood vessel. In our study sample the median amount of blood vessel rate for one subject was 4.8 with the ratio varying from 4 to 9 (shown on Figure 1).

An automated analytic protocol was used to calculate the oxygen saturation. All eligible vessels in all measured quadrants were used but physiological crossing of blood vessels were excluded manually from the evaluation due to the risk of mixing of the absorbance spectrum of the two types of blood vessels.

The machine type was Retinal Oximeter Oxymap T1 (Oxymap ehf. Reykjavik, Iceland) that was paired with the retinal camera TRC-50DX, (Topcon Corporation, Tokyo, Japan). For the analysis, an oxymap software (version 2.5.2; Oxymap Inc. Reykjavik, Iceland) was used. All investigated parameters included blood vessel oxygen saturation, differentiating arteries from veins. Blood vessel diameter was also noted.

The full details of the study protocol are described in Polidar et al. Biomed Pap Med Fac Univ Palacky Olomouc Czech Repub 2024 (16).

Mean values measured are noted in Table 2.

**Table 2:** Mean oximetry values in control and stenosis group.

**Parameter**	**Case**	**Control**
Arterial saturation (%)	97.6+/−3.3	97.3+/−2.3
Venous saturation (%)	62.5+/−5.7	59.7+/−7.4
AV-difference (%)	35.1+/−4.5	37.5+/−6.6
Arterial diameter (um)	112.6+/−11.5	110.2+/−11.5
Venous diameter (um)	161.5+/−21.8	159.0+/−18.2

## Statistical analysis

First, reliability of the carotid ultrasound was assessed using Spearman's non-parametric correlation coefficients.The level of significance was set to 0.05. Correlation coefficient for PSV to stenosis severity was 0.94 proving the reliability of the testing. However, no significant correlation was found between any ultrasonographic and oximetry parameters.

Reliability of the oximetry was secured by using the aforementioned standardized automated protocol. Then, the whole dataset was then visualized in dot-graph to evaluate the distribution of the data, observe possible effect of stenosis, comorbidities, retrograde blood flow and prior clinical symptoms.

In the first part of the study, paired testing was used comparing stenotic to non-stenotic sides in same patients to evaluate the reproducibility of the testing compared to Zhang et al. (10)

**Figure 2:**
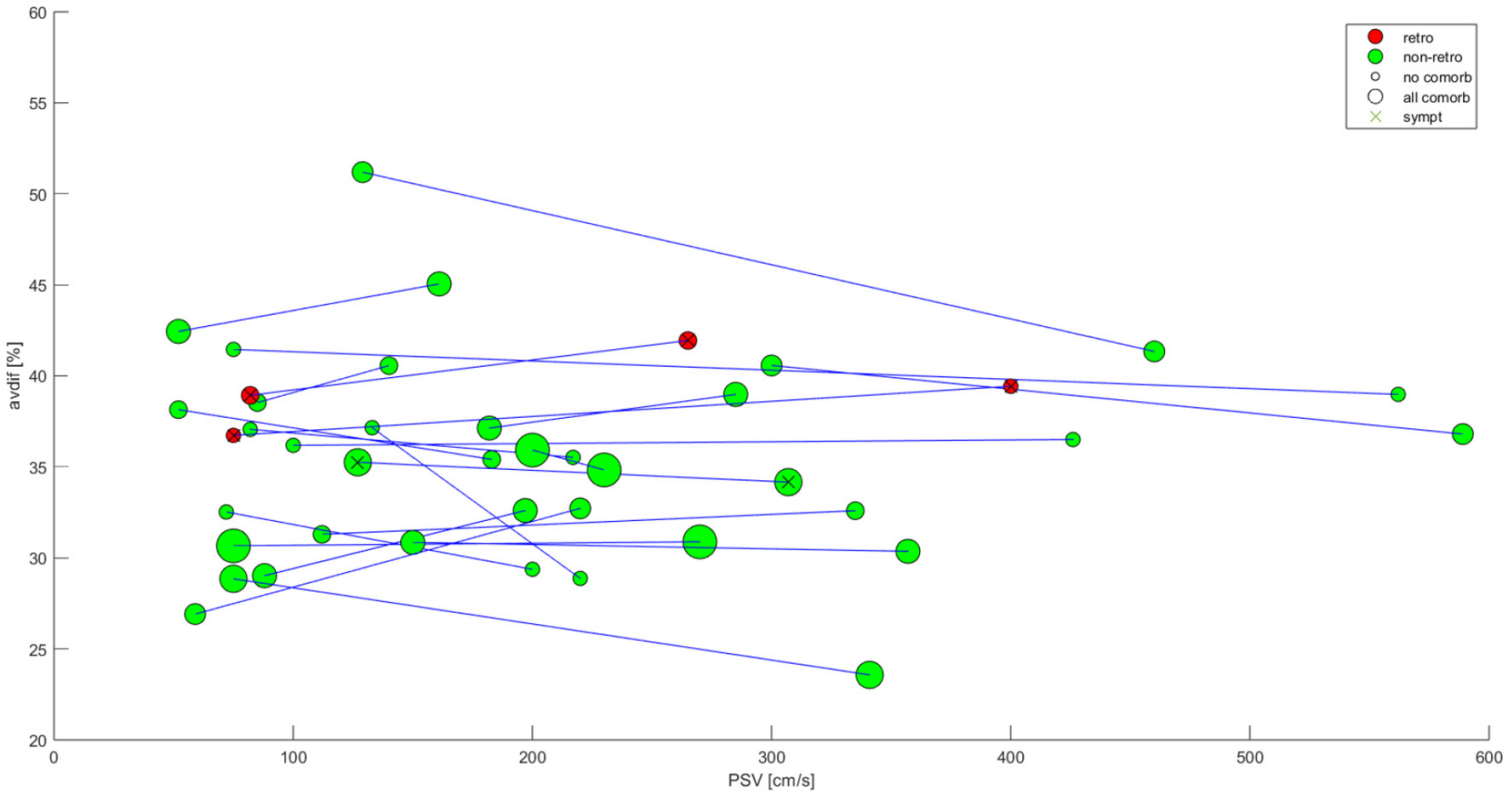
Paired analysis dot graph. X axis-PSV (cm/s); Y axis-AV difference (%). Green dots—ortograde ophtalmic blood flow in teh stenotic side, red dots—retrograde ophtalmic blood flow in the stenotic side, dot size—number of comorbidities. No significant correlation found, weak correlation in 2 retrograde ophtalmic blood flow patients.

For each patient, who underwent bilateral measurement, the AV- difference and the PSV was recorded on both sides. If the value of AV-difference was dependent on PSV, the slope of the line segments in Figure 2 should reveal a systematic pattern. Thus, we tested the slope of the line segments for significant deviation from zero (by means of the Wilcoxon Signed-rank test).

In second part of the study, we strived to evaluate the predictive value of AV-difference for the increasing stenosis severity We tried to build a multivariate linear regression model to explain AV-difference by means of the degree of stenosis and all available comorbidities. In the full model, no predictor was significant, so we sequentially excluded the predictors with the largest confidence intervals symmetrical around zero.

## Results

**Figure 3:**
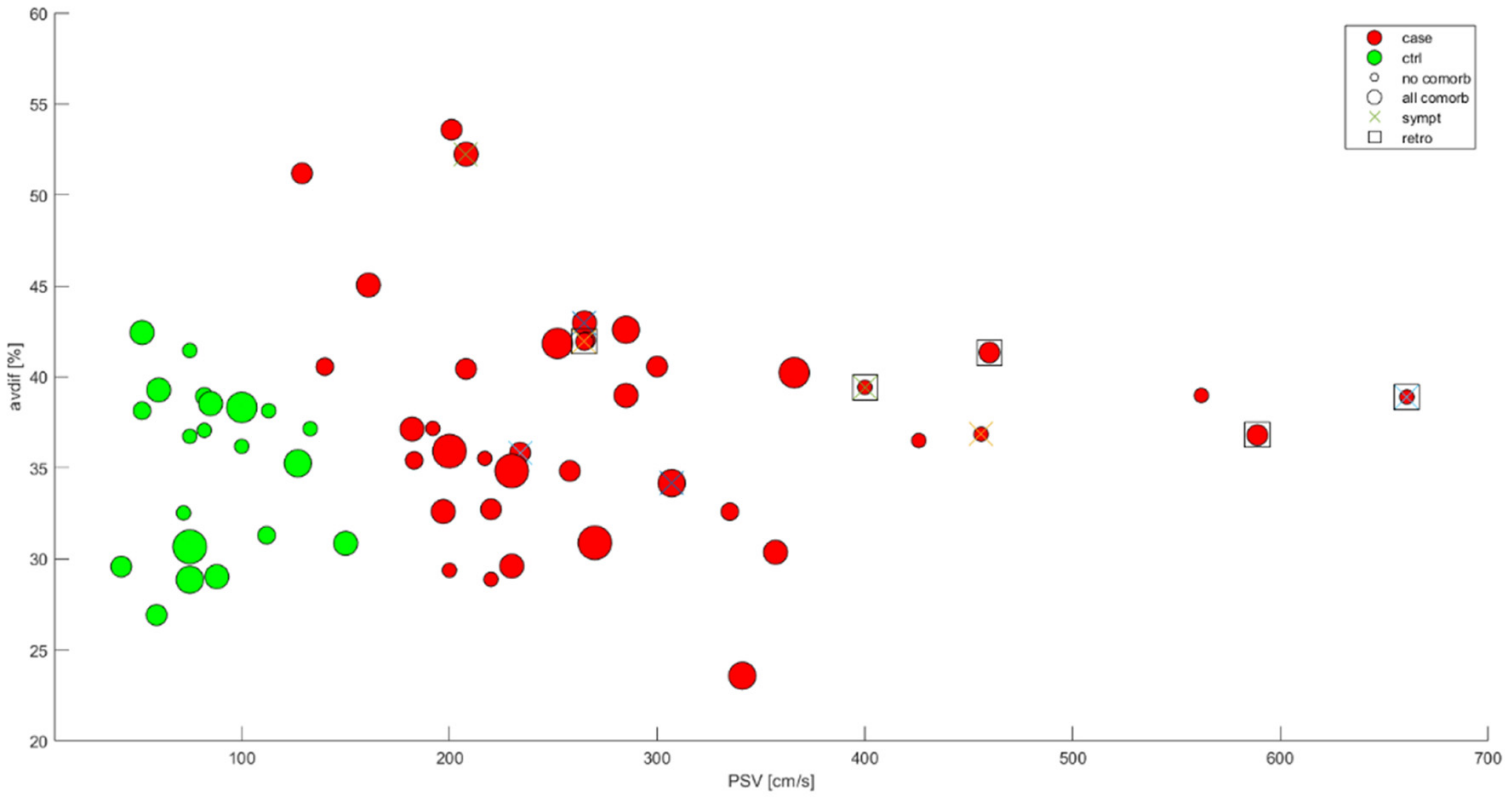
Data distribution dot graph. X axis-PSV (cm/s), Y axis-AV-difference (%). Controls-green, cases-red. Crossing-symptomatic (non-significant cerebrovascular event 6 months prior to the examination), dot size–number of comorbidities; square–retrograde ophtalmic blood flow No significant pattern observed in the baseline data distribution.

After assesing the reliability of the measurements, Dot graph presented by Figure 3 was used to visualize the distribution of the data. While differentiation between cases and controls via carotid ultrasound was apparent, no apparent relationship between stenosis severity and AV difference was noted. AV difference was not affected by the distribuition of comorbidities or clinical symptoms. Retrograde ophtalmic blood flow was observed in higher severity stenoses with higher PSV values.

The data collected were summarized in Figure 3.

Paired analysis of the 16 patients eligible for contributing to both control and stenotic group was performed using Wilcoxon Sign rank paired test. Visual representation of those patients is presented in dot graph in Figure 2.

The test does not reject the hypothesis that the median of the slopes is zero (p = 0.72). Thus, no statistically significant dependence of AV-difference and PSV was found in patients with measurement of both sides. This type of analysis takes into account all the comorbidities as the two measurements naturally belong to the same patient.

Apart from 2 patients with retrograde ophtalmic blood flow, no significant change in AV difference was observed. These results differ greatly from Zhang et al. (10) where significant increase in AV difference was noted.

In the second part of the statistical testing, multivariate linear regression analysis was built to predict the relationship between AV difference and stenosis severity in the context of comorbidities. After excluding the comorbidities with no significant effect on the AV difference distribution three predictors remained in the final model, all of the reaching statistical significance forming final equation.


$$\begin{array}{c} \text{AV difference}=35+0.04\;*\text{ stenosis }3.2\;*\;\left(\text{atherosclerosis}==1\right)\\ -4.7\;*\;\left(\text{atrial fibrillation}=1\right) \end{array}$$

The interpretation of the model is the following. For patients without atherosclerosis, without fibrillation, and without stenosis, the expected level of AV-difference is 35%. AV-difference increases by ~0.4 percent for every 10 percent of increase in stenosis. Atherosclerosis increases AV-difference by about 3% (for a given level of stenosis). Fibrillation, interestingly seems to have a protective effect—the presence of fibrillation decreases AV-difference by about 5% (for a given level of stenosis).

The linear model is neither very good (residuals are high), nor appropriate (the dependent variable is in percent). However, given the low number of patients are high number of predictors, it has a hypothesis generating potential.

## Discussion

Our primary aim was not fulfilled. We did not prove significant correlation between the stenosis severity and increased oxygen extraction. Even after comorbidity factor elimination by using paired statistical analysis in group of 16 patients, comparing pairs of stenotic and non-stenotic blood vessels, the results remained insignificant.

From the predictions of the second part it can be observed that while AV difference has a trend of slight increase with the stenosis severity, the individual factors create strong bias proving this method unreliable for evaluating the stenosis severity only. From the comorbidities, surprisingly only systemic atherosclerosis and atrial fibrillation showed some impact. The connection between atrial fibrilation and retinal oximetry parameters has not been studied yet and these results should be interpreted very carefully. For the systemic atherosclerosis, in our study it was designed as a presence of any cardiovascular event other than ischaemic stroke.

It is clear that the effects of both stenosis and systemic comorbidities influence the retinal oximetry, however their true extent remains unpredictable and combination of all those factors may contribute to the retinal oxygen metabolism.

Such results differ greatly from Zhang et al. (10). There are some possible reasons.

First-the study was conducted on Caucasian population instead of Chinese, which may cause different aspects of blood circulation and cardiovascular comorbidities. This might abide for the different results using paired testing when systemic comorbidities are eliminated.

Second-There are a number of systemic diseases that impact the amount of oxygen in the blood such as chronic obstructive pulmonary disease, congestive heart failure; or the affinity of hemoglobin for oxygen as in diabetes mellitus. Some neurological diseases such as Alzheimer disease and multiple sclerosis may also influence the retinal oxygen demands altering the results of the oximetry (17–21). Other cardiovascular risk factors such as atrial fibrillation, smoking, alcoholism, dyslipidaemia or hypertension may influence blood vessel wall structure and change the oxygenation capacity of the supplied tissue.

We presented the prevalence of relevant diseases in Table 1. However, we cannot compare the disease prevalence with Zhang et al., since it wasn't included in the text. Furthermore, we have to consider that only a portion of patients is presented with a known diagnosis. Even in our small study sample some patients might have not been diagnosed yet. Furthermore the extent of the disease progression may result in adaptive changes influencing the retinal oxygen metabolism as well.

Third-as mentioned before, are certain limits to ultrasonographic estimation of the stenosis severity due to the physical laws concerning blood flow and interindividual variability. While these factors should not play a significant role when examining 2 sides of 1 patient, in the real population when bilateral testing is unavailable the situation might be more complicated.

When these limitations are combined, we can say that retinal oximetry is not suitable for the evaluation of the carotid stenosis severity itself due to the presence of various factors limiting its use. There may be unilateral contraindications for retinal oximetry evaluation (in our study sample 14 eye-artery pairs out of 70 were not eligible for oximetry evaluation at all), uncertainty of ultrasonographic estimation of the stenosis severity in lower degree stenosis and considerable influence of diagnosed and maybe underdiagnosed systemic comorbidities which can be only eliminated by using the paired testing.

However, based on the aforementioned assumptions this method could still provide valuable data. Real world evidence shows that a combination of factors such as stenosis severity, atherosclerotic and cardiovascular risk factors influence the overall risk of stroke greatly. Retinal oximetry yields results that are influenced by all of those together. We might say that the possible predictive value of retinal oximetry would not lie in evaluating the stenosis severity and its direct impact on oxygen metabolism in the retina itself, rather than estimating the global risk of stroke. More research in the field is needed however to potentially prove that assumption.

## Data Availability

The datasets presented in this study can be found in online repositories. The names of the repository/repositories and accession number(s) can be found in the article/Supplementary material.
